# Enhanced Production of Bacterial Cellulose in *Komagataeibacter xylinus* Via Tuning of Biosynthesis Genes with Synthetic RBS

**DOI:** 10.4014/jmb.2006.06026

**Published:** 2020-07-06

**Authors:** Dong Hoon Hur, Woo Sung Choi, Tae Yong Kim, Sang Yup Lee, Jin Hwan Park, Ki Jun Jeong

**Affiliations:** 1 Department of Chemical and Biomolecular Engineering, BK21 Plus Program, Korea Advanced Institute of Science and Technology (KAIST), Daejeon 34141, Republic of Korea; 2 Biomaterials Lab, Samsung Advanced Institute of Technology (SAIT), Samsung Electronics Co., Ltd., Suwon 16678, Republic of Korea; 3 KAIST Institute for the Bi°Century, Korea Advanced Institute of Science and Technology (KAIST), Daejeon 34141, Republic of Korea

**Keywords:** Bacterial cellulose, *Komagataeibacter xylinus*, synthetic RBS, fluorescence-activated cell sorting

## Abstract

Bacterial cellulose (BC) has outstanding physical and chemical properties, including high crystallinity, moisture retention, and tensile strength. Currently, the major producer of BC is *Komagataeibacter xylinus*. However, due to limited tools of expression, this host is difficult to engineer metabolically to improve BC productivity. In this study, a regulated expression system for *K. xylinus* with synthetic ribosome binding site (RBS) was developed and used to engineer a BC biosynthesis pathway. A synthetic RBS library was constructed using green fluorescent protein (GFP) as a reporter, and three synthetic RBSs (R4, R15, and R6) with different strengths were successfully isolated by fluorescence-activated cell sorting (FACS). Using synthetic RBS, we optimized the expression of three homologous genes responsible for BC production, *pgm*, *galU*, and *ndp*, and thereby greatly increased it under both static and shaking culture conditions. The final titer of BC under static and shaking conditions was 5.28 and 3.67 g/l, respectively. Our findings demonstrate that reinforced metabolic flux towards BC through quantitative gene expression represents a practical strategy for the improvement of BC productivity.

## Introduction

Cellulose is the most abundant biopolymer in the world and is easily found in plants. In addition, various microorganisms, including bacteria, algae, and fungi, produce cellulose. Among bacteria, *Komagataeibacter xylinus* (previously known as *Gluconacetobacter xylinus* or *Acetobacter xylinum*) is a primary producer of bacterial cellulose (BC) [1, 2]. BC has outstanding properties, such as high water retention value, surface area, crystallinity, biodegradability, biocompatibility, and high purity due to the absence of lignin and hemicellulose [3-6]. Due to these properties, BC has potential applications in the biomedical, cosmetic, automotive, packaging, health food, electrical, and sensor industries, with a rapidly increasing commercial value [7-9].

**Fig. 1:**
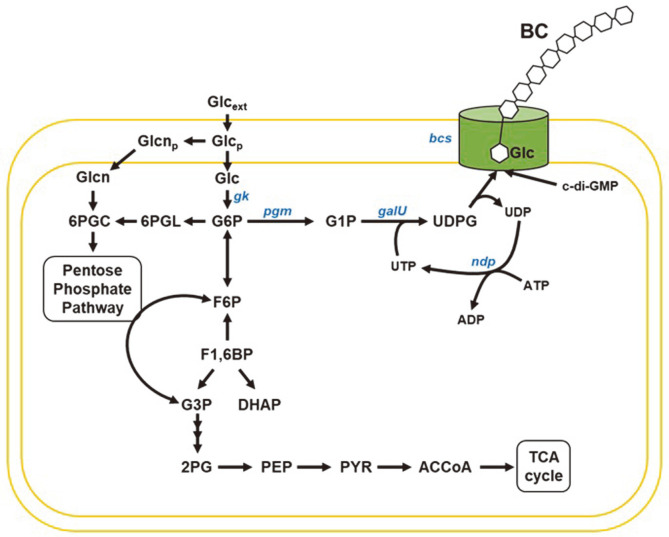
Metabolic pathway of *K. xylinus* DSM 2325 and the BC biosynthesis pathway. Metabolite abbreviations are: Glc_ext_, D-glucose at extracellular medium; Glc_p_, D-glucose at periplasm; Glc, D-glucose; G6P, D-glucose-6-phosphate; G1P, D-glucose-1-phosphate; UDPG, UDP-glucose; c-di-GMP, cyclic diguanylic acid; Glcn_p_, D-gluconate at periplasm; Glcn, D- gluconate; 6PGC, 6-phospho-D-gluconate; 6PGL, 6-phospho-D-glucono-1,5-lactone; F6P, D-fructose-phosphate; F1,6BP, D- fructose-1,6-bisphosphate; DHAP, dihydroxyacetone phosphate; G3P, D-glyceraldehyde-3-phosphoate; 2PG, 2-phospho-D- glycerate; PEP, phosphoenolpyruvate; PYR, pyruvate; ACCoA, acetyl-CoA.

As industrial demand for BC increases, many efforts have been made to improve its productivity. Most of the research has focused on the optimization of bioprocesses, including culture conditions and medium compositions, among others [10]. However, due to a limited understanding of the BC biosynthesis mechanism and a lack of efficient genetic tools, there have been few attempts to engineer host organisms for enhanced BC production [2, 8, 9]. Using glucose as a main carbon source of cellulose, *K. xylinus* synthesizes BC by expressing glucokinase (*gk*), phosphoglucomutase (*pgm*), UTP-glucose-1-phosphate uridylyltransferase (*galU*), and bacterial cellulose synthase (*bcsABCD*) (Fig. 1). The expression of the *bcs* operon (*bcsABCD*) can be used to increase the production of BC. Mangayil *et al*. reported that the overexpression of *bcsABCD* in *K. xylinus* DSM 2325 resulted in a production of BC as high as 4.3 g/l, which represents a 10.75-fold increase of BC produced by the wild-type strain [2]. In addition, recent advances in genome-scale metabolic modeling of BC-producing hosts have highlighted the re-design of metabolic pathways to reinforce the metabolic flux toward BC biosynthesis [11]. This systematic engineering can be further improved by fine-tuning gene expression in several critical steps, as the highest productivity is not achieved under the highest level of gene expression but rather under optimal levels. Recently, two research groups reported on the development of genetic toolkits, including synthetic promoters, RBSs, and plasmids useful for tuning the expression of target genes in *Komagataeibacter* spp. [12, 13]. Through the combinatorial assembly of these synthetic toolkits, the production of BC can be increased further, and novel cellulose-based materials can be created. For the reliable and precise control of gene expression, more synthetic parts need to be developed [14-16]. However, the development of fully-synthetic toolkits for the metabolic engineering of *Komagataeibacter* spp. via high-throughput screening covering numerous sizes of libraries, has not yet been reported.

In this study, we engineered BC-producing *K. xylinus* using synthetic RBSs capable of tunable gene expression. We constructed a random library of RBSs using GFP as a reporter gene, and three potential RBSs of various strengths were isolated using FACS-based high-throughput screening. The usefulness of the synthetic RBSs was verified, and they were then employed to enhance BC productivity by reinforcing metabolic flux towards BC biosynthesis.

## Materials and Methods

### Bacterial Strains, Media, and Culture Conditions

All of the bacterial strains used in this study are listed in Table 1. *Escherichia coli* XL1-Blue was used for cloning and library construction. *E. coli* were cultivated in Luria-Bertani (LB) medium (10 g/l tryptone, 5 g/l yeast extract, 10 g/l NaCl) and chloramphenicol (Cm, 35 mg/l) was supplemented as a sole antibiotic. *K*. *xylinus* DSM 2325 was purchased from the DSMZ (German Collection of Microorganisms and Cell Cultures) and used for RBS screening and BC production. *K. xylinus* was cultivated in Hestrin-Schramm (HS) medium (50 g/l glucose, 5 g/l peptone, 5 g/l yeast extract, 2.7 g/l anhydrous disodium phosphate, and 1.15 g/l citric acid monohydrate) [17,18]. For the RBS library screening, cells were cultivated in HS-Cel medium containing 0.4% (v/v) cellulase from *Trichoderma reesei* (Sigma-Aldrich Co., USA). For BC production, 1% (v/v) ethanol was added to the HS medium. Cells were incubated at 30°C for up to 5 days in a static incubator or under shaking (230 rpm) conditions in either 1 – 2 ml medium in a 14 ml round-bottom culture tube, 25 ml medium in a 125 ml flask for shaking culture or 25 ml medium in 90 (d) × 15 (h) mm Petri dish for static culture. For precise comparison of BC productivity, initial OD_600_ values were set to 0.02 for all BC production experiments. Seed cultures were carried out in HS-Cel media, and washed with fresh HS medium before transferring to main culture. BC production was carried out for 120 h in fresh HS medium containing 1% (v/v) ethanol. In all cultivation of recombinant *K. xylinus*, 140 mg/l of chloramphenicol was added as a sole antibiotic.

### Plasmid Manipulation and Construction of Synthetic RBS Library in *K. xylinus*

All plasmids used in this study are listed in Table 1. The polymerase chain reaction (PCR) was carried out using a C1000 Thermal Cycler (Bio-Rad, USA) and PrimeSTAR HS Polymerase (TAKARA BIO, Inc., Japan). Primer nucleotide sequences used for PCR are listed in Table S1. As a positive control for the RBS screening, we constructed a GFP expression system (pDHJC_sfGFP) by replacing mRFP1 gene with sfGFP and rrnBT1 terminator with a strong terminator, ECK120033736 [13], in J23104-mRFP1-331Bb [12]. For construction of the RBS library, a DNA fragment including a fully randomized RBS sequence (12 bp) and sfGFP was amplified by PCR with primers Fw_Lib and Rv_sfGFP. The PCR product was digested with *Spe*I and *Xho*I restriction enzymes, and cloned into the same restriction enzyme sites of pDHJC_sfGFP. The ligated plasmids were transformed with *E. coli* XL1-Blue by electroporation. The synthetic RBS library in *E. coli* was purified with Hybrid-Q Plasmid Rapidprep (Geneall, Korea) and then retransformed into *K. xylinus* by electroporation. Transformed *K. xylinus* cells were recovered in HS-Cel media containing cellulase for 6 h and spread on HS agar plates containing 140 mg/l of chloramphenicol at 30°C for 48 h. All of the grown recombinant *K. xylinus* colonies were collected using 300 ml of HS-Cel media and cultured at 30°C and 230 rpm to degrade cellulose and make the library homogenous. After 1 h, the recombinant *K. xylinus* cells were stored at -80°C as 15% glycerol stocks. For the expression of *pgm*, *galU*, and *ndp* genes with three isolated RBSs, each gene was amplified from chromosomal DNA of *K. xylinus* by PCR with the primer sets listed in Table S1. After the amplification of *pgm*, *galU*, and *ndp*, each PCR product was digested with BbsI and BsaI restriction enzymes, and cloned into the NdeI and XhoI sites of pDHJCR4_sfGFP, pDHJCR6_sfGFP and pDHJCR15_sfGFP, yielding pDHJCR4_pgm, pDJHCR4_galU, pDHJCR4_ndp, pDHJCR6_pgm, pDHJCR6_galU, pDHJCR6_ndp, pDHJCR15_pgm, pDHJCR15_galU and pDHJCR15_ndp. For the expression of all *pgm*, *galU* and *ndp* genes in a single plasmid with R15 RBS, vector backbone was amplified using primers Fw_GibV_pDHJCR15 and Rv_GibV_pDHJCR15 using pDHJCR15_sfGFP as a template. A *pgm* gene was amplified using primers Fw_Gib1_R15_pgm and Rv_Gib1_pgm using pDHJCR15_pgm as a template, and a *galU* gene was amplified using primers Fw_Gib2_R15_galU and Rv_Gib2_galU using pDHJCR15_galU as a template. An *ndp* gene was amplified using primers Fw_Gib3_R15_ndp and Rv_Gib3_ndp using pDHJCR15_ndp as a template. All PCR products were mixed and ligated to prepare pDHJCR15_pgm_galU_ndp using Gibson Assembly Master Mix (New England Biolabs, Ipswich, MA, USA) following the manufacturer’s manual.

### Library Screening Using FACS

The synthetic RBS library of *K. xylinus* was cultivated in HS-Cel media at 30°C for 48 h with shaking (230 rpm). The fully grown cells were then transferred into fresh HS-Cel media at a final concentration of 1% (v/v), and further cultured at 30°C and 230 rpm for 16 h. The cells were then screened using FACS (MoFlo XDP; Beckman Coulter, Inc., USA) based on fluorescence intensity detection with a 488 nm laser and a 530/40 band-pass filter for the sfGFP emission spectrum. The cells with the greatest fluorescence intensity (top 1%) were sorted and directly inoculated to fresh HS-Cel media, followed by culturing for 48 h. Then, the enriched microbial cultures were transferred into fresh HS-Cel media for the following round of FACS sorting. Starting from the 5th round of sorting, individual clones were randomly selected and inoculated into HS-Cel media for individual analysis of fluorescence intensity. After 48 h of cultivation, pre-cultured cells were transferred into the main culture and cultivated for 16 h. The fluorescence intensity of each clone was analyzed using FACS.

During the 1^st^ to 3^rd^ rounds of sorting, the ‘purify’ mode was used to isolate only single droplets containing only fluorescent cells without any negative cells [14]. Using the purify mode, the quality of cell sorting could be highly increased, although with a large fraction of loss. Thus, during the 1^st^ round of sorting, more than 1,000,000 cells were sorted, while 500,000 cells were sorted during the 2^nd^ and 3^rd^ rounds. During the 4^th^ and 5^th^ rounds, the ‘single cell’ mode was used to isolate only single cells separately from cell consortia. Although the loss was even higher than with the purify mode, 50,000 cells were considered to be enough for screening because the cells went through several sorting steps while the library size was 3 × 10^7^.

### BC Washing and Quantification

BC production was measured after several washing procedures to remove any impurities, including cell debris and salts, as described previously with some modifications [19]. The BC was washed twice with distilled water, once with 0.1 M NaOH solution, and once with distilled water. Each step was carried out using 125 ml of solution, with overnight incubation at 90°C. After washing, the BC was dried in an oven at 105°C for one week until completely dry. The dehydrated BC was quantified using a precision scale.

## Results and Discussion

### Synthetic RBS Library Construction and Screening

**Fig. 2:**
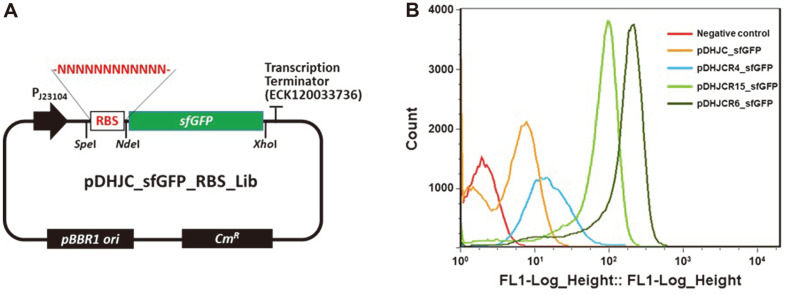
(A) Plasmid map of pDHJC_sfGFP_RBS_lib and (B) FACS analysis of isolated RBS clones. Totally 50,000 cells were counted for the analysis of negative control and pDHJC_sfGFP, and 100,000 cells were counted for the analysis of pDHJCR4_sfGFP, pDHJCR15_sfGFP and pDHJCR6_sfGFP.

For the construction of the RBS library, a 12 bp RBS region was designed with fully random nucleotide sequences (Fig. 2A). The synthetic RBS library was first constructed in *E. coli* XL1-Blue, and 3 × 10^7^ colonies were obtained. After the recovery of the library plasmids, these were re-transformed into *K. xylinus*, for which 2.5 × 108 colonies were obtained. After the cultivation of *K. xylinus* cells containing the RBS library, the cells with the high fluorescence intensity (FI) (top 1%) were sorted. In every round, the collected cells were inoculated into fresh HS- Cel media and cultured for the next round of sorting. During FACS screening, the increase of fluorescence intensity was clearly observed in every round (Fig. S1), which indicated the successful enrichment of cells with RBSs allowing for higher levels of gene expression. After the fifth round of sorting, the collected cells were cultured on HS-Cm agar plates for individual comparison. A total of 40 colonies were randomly picked and the FI in eac cell was analyzed using FACS (Fig. S2). Out of 40 clones, the RBS sequences in 20 clones showing different FIs were analyzed. Among them, 10 clones with high levels of FI (mean value, *M* = 548) were found to contain the same sequence (5’-TACACCGGAGAA-3’) (Table 2). An additional 7 clones with medium levels of FI (*M* = 215) contained the same sequence (5’-TAATGAGAGGCC-3’), while three clones with low levels of FI (*M* = 10) had the same sequence (5’-TTACAAAAATGA-30). As a result, three different RBSs were obtained, and named after their representative clone number: R6 (strong FI), R15 (medium FI), and R4 (weak FI) (Fig. 2B). Compared to the control RBS (*M* = 26) that was previously reported by Florea *et al*. [12], R4 exhibited slightly lower levels of FI, however, the FI levels of R6 and R15 were much higher (21- and 8.3-fold increase, respectively).

### Characterization of Isolated Synthetic RBSs

In gene expression, the translation rate is highly dependent on the thermodynamic structure of the 5’ untranslated region (5’ UTR), including the RBS sequence. Based on this thermodynamic analysis, the strength of the isolated RBS can be predicted [20, 21]. To understand the strength of the three isolated RBSs, we performed thermodynamic analysis of each RBS using an untranslated region (UTR) designer [20]: 60 base mRNA sequences, including 25 bp of each 5’ UTR sequence and the first 35 bases of the sfGFP gene, were used for analysis. As a result, RBS R6 showed the most negative dG_UTR_ value, indicating a higher chance of ribosome binding and translation, while R15 showed a moderate dG_UTR_ value, and R4 showed a positive dG_UTR_ value, which correlated to the predicted expression levels (Fig. S3). The results of the thermodynamic analysis indicate an increasing trend in terms of strength in our RBS set.

### Expression of BC Synthesis Pathway Genes Using Synthetic RBSs and BC Productivity Under Shaking Condition

**Fig. 3:**
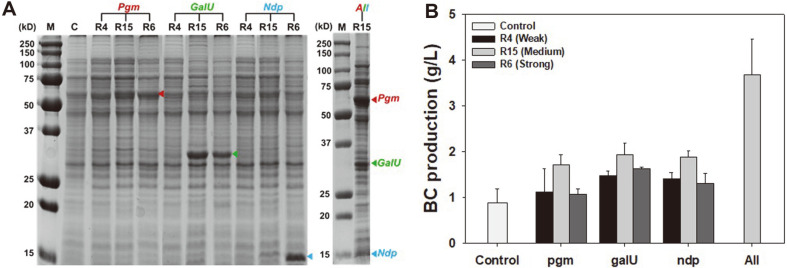
(A) SDS-PAGE analysis of wild type and recombinant *K. xylinus* expressing either *pgm*, *galU*, or *ndp* with each synthetic RBS (R4, R15 or R6) or expressing all three genes with R15 synthetic RBS (right). A wild- type *K. xylinus* without plasmid was used as a control (lane C), and *K. xylinus* harboring pDHJCR15_pgm_galU_ndp was used for expression of all genes. Red, green and blue arrowheads represent the band of Pgm, GalU, and Ndp, respectively. (B) BC productivity in recombinant *K. xylinus* after 120 h under shaking condition. Control represents a wild-type *K. xylinus* without plasmid.

Next, to enhance BC production in *K. xylinus*, the expression levels of the *pgm*, *galU*, and *ndp* genes, which are responsible for the production of BC, were examined using the newly isolated RBSs (R6, R15, and R4). The expression system of each gene was constructed with three different RBSs (a total of 9 sets). After cultivation in liquid HS medium, gene expression in each expression set was analyzed using SDS-PAGE. Among the three genes, the expression of the *ndp* gene exhibited high expression levels with strong R6 RBS, while *pgm* and *galU* exhibited higher expression levels with medium strength R15 (Fig. 3A). In each cultivation, the levels of BC production were also analyzed and compared. Interestingly, among the three RBSs, the use of medium strength R15 resulted in the highest production of BC: BC productivity expressing *pgm*, *galU*, and *ndp* under R15 was 1.71, 1.94, and 1.88 g/l, respectively, which represents a 1.9–2.2-fold increase compared to the *K. xylinus* wild type (Fig. 3B). In the case of *ndp* gene expression under strong R6 RBS, we found that most Ndp was produced in the form of insoluble aggregates (data not shown), which resulted in a lower production of BC (Fig. 3B).

Based on these results, we decided to use RBS R15 for the expression of all three genes, and constructed pDHJCR15_pgm_galU_ndp in which all three genes (*pgm*, *galU*, and *ndp*) were expressed together under PJ23104 promoter and RBS R15. During the cultivation of *K. xylinus* harboring pDHJCR15_pgm_galU_ndp, all three genes were found to be highly expressed with high solubilities (Fig. 3A). In addition, the BC production titer was also remarkably increased up to 3.67 g/l, which was 4.15-fold higher than the wild-type *K. xylinus* (Fig. 3B).

### BC Productivity Under Static Condition

Finally, to obtain film-like BC, static cultivation was conducted. During the cultivation of the engineered

**Fig. 4:**
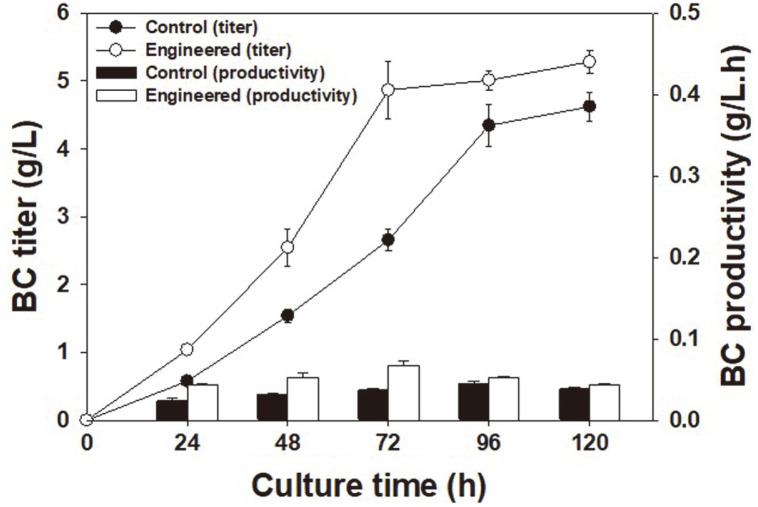
BC production in engineered *K. xylinus* harboring pDHJCR15_pgm_galU_ndp. Control represents a wild-type *K. xylinus*. Symbols: Closed and open circles represent BC titers in control and engineered *K. xylinus*, respectively. Black and white bars represent BC productivity of control and engineered *K. xylinus*, respectively.

*K. xylinus* strain, the BC titer increased rapidly, reaching up to 4.87 g/l at 72 h. During further cultivation (72 h to 120 h), the BC titer increased gradually up to 5.28 g/l (Fig. 4). Stagnant production of BC after 72 h may be caused by lowered mass transfer of O_2_, depletion of nutrients, cell death, pH drop, etc. [2]. During the cultivation of the control *K. xylinus* strain (wild type), the BC titer increased relatively slowly, reaching 4.62 g/l after 96 h. The maximum instantaneous BC productivity of the engineered strain was 67.6 mg/l/h at 72 h, while that of control strain was 48.1 mg/l/h at 96 h (Fig. 4). These results indicated that our engineered strain was capable of more efficient, faster, and higher production of BC.

## Conclusion

In this study, we constructed a fully synthetic RBS library and successfully isolated novel synthetic RBSs with different strengths in *K. xylinus* cells using FACS screening. Using the synthetic RBSs, the expression levels of three genes (*pgm*, *galU*, and *ndp*) involved in the biosynthesis of BC were optimized, and by reinforcing the metabolic pathway towards BC production, the enhanced production of BC could be achieved under both static and shaking culture conditions. To the best of our knowledge, this is the first study to engineer *K. xylinus* with synthetic parts to enhance the BC biosynthesis via high-throughput screening strategy. As we clearly demonstrated here (particularly in Fig. 3), the use of a stronger expression system does not always ensure higher production, although the balancing of expression level is more critical in multiple gene expressions to achieve higher production. For finer tuning of each gene expression, more synthetic parts including promoters, terminators, and regulators need to be further developed, and their use in the engineering of the BC biosynthesis pathway can serve to establish *K. xylinus* as a potential host for increased production of BC. Previously, we succeeded in engineering IS-element- resistant strain (*K. xylinus* SAIT-IS) by modifying a putative IS-element recognition sequence in a *bcsA* gene, which resulted in 1.7-fold higher productivity [18]. We also believe that the introduction of the present optimized system (pDHJCR15_pgm_galU_ndp) with more synthetic parts in the engineered strain can provide a synergetic effect for BC production.

## Supplemental Materials



Supplementary data for this paper are available on-line only at http://jmb.or.kr.

## Figures and Tables

**Table 1:** Bacterial strains and plasmids used in this study.

	Characteristics	Ref. or source
Strains	
XL1-Blue	*recA1 endA1 gyrA96 thi-1 hsdR17 supE44 relA1 lac [F′ proAB lacI^q^ZDM15 Tn10 (Tet^r^)]*	Stratagene^a^
*K. xylinus*	Wild type	DSM 2325
Plasmids	
J23104-mRFP1-331Bb	pSEVA331 derivative; P_J23104_, Control RBS	[12] Addgene^b^
pDHJC_sfGFP	J23104-mRFP1-331Bb derivative; ECK120033736 terminator, sfGFP	This study
pDHJC_sfGFP_RBS_lib	pDHJC_sfGFP derivative; sfGFP, RBS library	This study
pDHJCR4_sfGFP	pDHJC_sfGFP derivative; sfGFP, RBS R4	This study
pDHJCR15_sfGFP	pDHJC_sfGFP derivative; sfGFP, RBS R15	This study
pDHJCR6_sfGFP	pDHJC_sfGFP derivative; sfGFP, RBS R6	This study
pDHJCR4_pgm	pDHJC_sfGFP derivative; *pgm*, RBS R4	This study
pDHJCR4_galU	pDHJC_sfGFP derivative; *galU*, RBS R4	This study
pDHJCR4_ndp	pDHJC_sfGFP derivative; *ndp*, RBS R15	This study
pDHJCR15_pgm	pDHJC_sfGFP derivative; *pgm*, RBS R15	This study
pDHJCR15_galU	pDHJC_sfGFP derivative; *galU*, RBS R15	This study
pDHJCR15_ndp	pDHJC_sfGFP derivative; *ndp*, RBS R15	This study
pDHJCR6_pgm	pDHJC_sfGFP derivative; *pgm*, RBS R6	This study
pDHJCR6_galU	pDHJC_sfGFP derivative; *galU*, RBS R6	This study
pDHJCR6_ndp	pDHJC_sfGFP derivative; *ndp*, RBS R6	This study
pDHJCR15_pgm_galU_ndp	pDHJC_sfGFP derivative; *pgm*, *galU*, *ndp*, RBS R15	This study

^a^Stratagene (La Jolla, CA, USA)

^b^Addgene (Watertown, MA, USA)

**Table 2:** Nucleotide sequences of isolated RBS and corresponding GFP fluorescence intensities.

RBS	Sequence (5′ to 3′)	Mean fluorescence intensity (*M*)
R6	TACACCGGAGAA	548
R15	TAATGAGAGGCC	215
R4	TTACAAAAATGAT	10
Control^a^	GAAAGAGGAGAAA	26

^a^ref 12
