# Supplementary material for: Enhanced Production of Bacterial Cellulose in Komagataeibacter xylinus Via Tuning of Biosynthesis Genes with Synthetic RBS
Source: J Microbiol Biotechnol. 2020 Jul 6;30(9):1430–5. doi: 10.4014/jmb.2006.06026 (PMC9728265; doi:10.4014/jmb.2006.06026)
Supplement: Supplementary file 1 [file JMB-30-9-1430-supple.pdf]

https://doi.org/10.4014/jmb.2006.06026

Supplementary Table S1. List of PCR primers used in this study.

Primer name	Primer sequences (5′ to 3′)
Fw_sfGFP	TGCTAGCTACTAGTGAAAGAGGAGAAATACCATATGCA TCACCATCACCATCACATGAGCAAAGGAGAAGAACTTT
Rv_sfGFP-ECK120033736	ATGACTCTGCAGAACGCATGAGAAAGCCCCCGGAAGAT CACCTTCCGGGGGCTTTTTTATTGCCTCGAGTTATTATTT
Fw_Lib	CTAGCTACTAGTNNNNNNNNNNNNTACCATATGCATCA CCATCACC
Rv_sfGFP	AGCGCTCGAGTTATTATTTGTAGAGCTCATCCATGCC
Fw_Seq_pDHJC	AGGCCCAGTCTTTCGACTG
Rv_Seq_pDHJC	TATCAACGGTGGTATATCCAGTGA
Fw_pgm	ACACACGAAGACCATATGCCCAGCGTAAGCCCTTT
Rv_pgm	GTGTCTCGAGTTATCAATGGTGATGGTGATGGTGCTTCT GCGCAGCCTTGGCAAA
Fw_galU	ACACGGTCTCATATGATTAAGCCCCTTAAAAAAGCCGTA TTG
Rv_galU	GTGTGGTCTCCTCGAGTTATCAATGGTGATGGTGATGGT GTTTATACTTTTTGAGGAATTCCCGCACG
Fw_ndp	ACACGGTCTCATATGGCAGTCGAACGTACCCTCTCCATC
Rv_ndp	GTGTGGTCTCCTCGAGTTATCAATGGTGATGGTGATGGT GGGGCAGGATCTCGGTGCCGG
Fw_GibV_pDHJCR1	CTCGAGGCAATAAAAAAGCCCCCG
Rv_GibV_pDHJCR15	CTATCGTCCTAGTCCCTACTGCTAGCACAATACCTAGGA CTGAGCTAG
Fw_Gib1_R15_pgm	AGTAGGGACTAGGACGATAGTACTAGTTAATGAGAGGC CTACCATATGCCCAG
Rv_Gib1_pgm	TGACGTGTAACTCGATTGATCTCGAGTTATCAATGGTGA TGGTGATGGTGC
Fw_Gib2_R15_galU	ATCAATCGAGTTACACGTCATACTAGTTAATGAGAGGCC TACCATATGATTAAGCCCC
Rw_Gib2_galU	CACTAGTTCCGAGACTGTACCTCGAGTTATCAATGGTGA TGGTGATGGTGTTTATACTTT
Fw_Gib3_R15_ndp	GTACAGTCTCGGAACTAGTGTACTAGTTAATGAGAGGCC TACCATATGGCAGTCG
Rv_Gib3_ndp	ACCTTCCGGGGGCTTTTTTATTGCCTCGAG

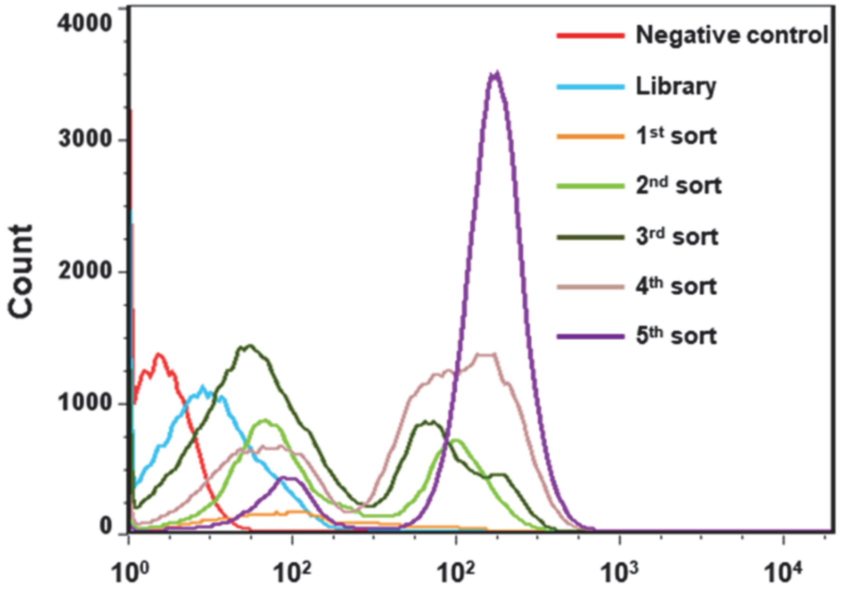

Supplementary Figure S1. FACS analysis of recombinant *K. xylinus* harboring RBS library. Totally 50,000 cells were counted for the analysis of negative control, library, 1st sort and 2nd sort, and 100,000 cells were counted for the analysis of 3rd sort, 4th sort and 5th sort.

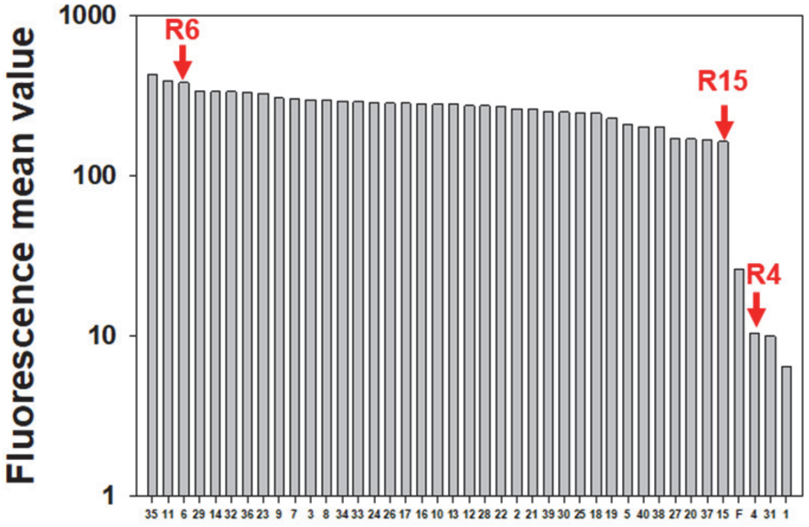

Supplementary Figure S2. Fluorescence mean values of single recombinant *K. xylinus* clones harboring RBS library from the FACS analysis

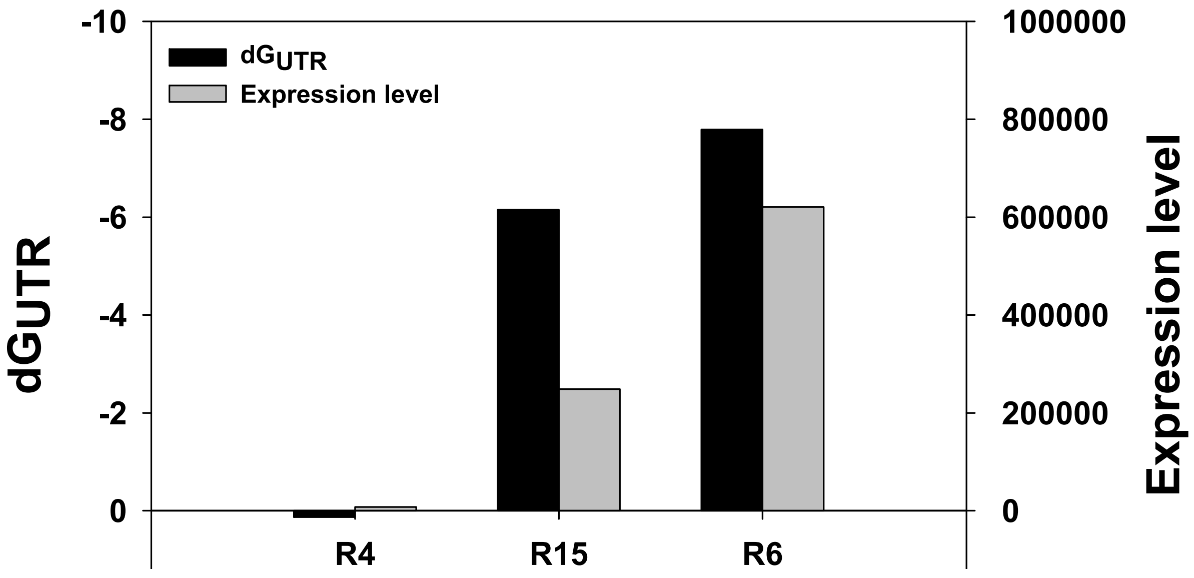

Supplementary Figure S3. Thermodynamic analysis of newly isolated RBSes and sfGFP gene
